# Identification and Functional Evaluation of a Novel *TBX4* Mutation Underlies Small Patella Syndrome

**DOI:** 10.3390/ijms23042075

**Published:** 2022-02-14

**Authors:** Ping Li, Wenli Lan, Jiaying Li, Yanping Zhang, Qiuhong Xiong, Jinpei Ye, Changxin Wu, Han Xiao

**Affiliations:** Institutes of Biomedical Sciences, The Key Laboratory of Chemical Biology and Molecular Engineering of Ministry of Education of China, The Key Laboratory of Medical Molecular Cell Biology of Shanxi Province, Shanxi University, Taiyuan 030006, China; lanwenli1998@163.com (W.L.); Jiaying148@126.com (J.L.); 201923105006@email.sxu.edu.cn (Y.Z.); qxiong@sxu.edu.cn (Q.X.); jinpei.ye@sxu.edu.cn (J.Y.); cxw20@sxu.edu.cn (C.W.)

**Keywords:** *TBX4* mutation, small patella syndrome, *FGF10*, mesenchymal stem cell

## Abstract

Small patella syndrome (SPS) is a rare autosomal dominant disorder caused by mutations in *TBX4* gene which encodes a transcription factor of *FGF10*. However, how *TBX4* mutations result in SPS is poorly understood. Here, a novel *TBX4* mutation c.1241C>T (p.P414L) was identified in a SPS family and series of studies were performed to evaluate the influences of *TBX4* mutations (including c.1241C>T and two known mutations c.256G>C and c.743G>T). Results showed that mesenchymal stem cells (MSCs) with stable overexpression of either *TBX4* wild-type (*TBX4*^wt^) or mutants (*TBX4*^mt^) were successfully generated. Immunofluorescence study revealed that both the overexpressed *TBX4* wild-type and mutants were evenly expressed in the nucleus suggesting that these mutations do not alter the translocation of TBX4 into the nucleus. Interestingly, MSCs overexpression of *TBX4*^mt^ exhibited reduced differentiation activities and decreased *FGF10* expression. Chromatin immunoprecipitation (ChIP) study demonstrated that TBX4 mutants still could bind to the promoter of *FGF10*. However, dual luciferase reporter assay clarified that the binding efficiencies of TBX4 mutants to *FGF10* promoter were reduced. Taken together, MSCs were firstly used to study the function of *TBX4* mutations in this study and the results indicate that the reduced binding efficiencies of TBX4 mutants (TBX4^mt^) to the promoter of *FGF10* result in the abnormal biological processes which provide important information for the pathogenesis of SPS.

## 1. Introduction

*Tbx* genes are a group of highly conserved genes among vertebrates, which play an important role in the formation and development of mesoderm in vertebrates [1,2]. T-box transcription factor 4 (*TBX4*) gene belongs to the T-box transcription factor family and is located on the human 17q23.2 chromosome [3]. It consists of eight exons and encodes a transcription factor which is expressed in hindlimb buds and plays a critical role in the developmental regulation of hindlimbs during the embryonic stage [2,4,5].

The importance of the *TBX4* gene in the developmental pathways of the lower limbs and the pelvis in humans were established based on the identification of heterozygous *TBX4* mutations in small patella syndrome (SPS; OMIM 147891) patients, and the observation of similar skeletal phenotype on animals lacking *Tbx4* gene [6,7].

T-box family contains a DNA binding domain (about 180 amino acids), which is highly conserved across different species. However, no other similar sequence fragment was found among different *T-box* family members, suggesting that the DNA binding domain is crucial and any change may cause functional defect results in human developmental diseases [8]. The typical feature of SPS caused by *TBX4* mutation is patellar aplasia or hypoplasia, and patients are often accompanied by abnormalities of pelvis and femur. In the lower limbs, the space between the first and second toes is widened and both fourth and fifth toes are often shortened, and even some patients may be accompanied with flat feet [7]. In addition, *TBX4* mutation can lead to childhood onset pulmonary arterial hypertension (PAH) with common clinical features including pulmonary capillary dysplasia, acinar dysplasia, respiratory failure, and in severe cases, death [9]. A large number of studies have shown that *Tbx4*^−/−^ embryos die by E10.5 and *Tbx4* deficiency affects the outgrowth of hindlimb indicating a key role of *Tbx4* in the formation of the hindlimb [10,11,12].

*FGF10*, fibroblast growth factor, plays an important role in the embryonic development, cell proliferation, cell differentiation and branching morphogenesis [13,14]. Moreover, the production of *Fgf10* is regulated by Tbx4 in chicken and mouse mesenchymal cells [15,16]. As a transcription factor, TBX4 induces the occurrence and formation of hindlimb buds through the activation of *Fgf10* expression [17]. Further, *Tbx4* interference leads to the inhibition of *Fgf10* expression and failure formation of lung bud in animal models [15]. Recent studies have shown that TBX4-FGF10-FGFR2 epithelial-stromal signal transduction pathway plays an important role in human lung organogenesis [18]. *TBX4* mutation leads to SPS and PAH, whereas *FGF10* mutation results in acinar dysplasia (AcDys) [19], indicating the pivotal roles of TBX4-FGF10 in the developmental regulation of hindlimb and lung. SPS causing from *TBX4* mutation is supposed due to the loss-of-function of TBX4, but no experimental evidence supports the pathogenesis of the reported *TBX4* mutations [20]. The aim of this study was to characterize the pathogenicity of one novel mutation c.1241C>T (p.P414L) and two known mutations c.256G>C (p.E86Q) and c.743G>T (p.G248V) in *TBX4* and illustrate the potential molecular mechanism of the *TBX4* mutations resulting in SPS.

## 2. Results

### 2.1. Identification of a Novel TBX4 Missense Mutation

**Figure 1:**
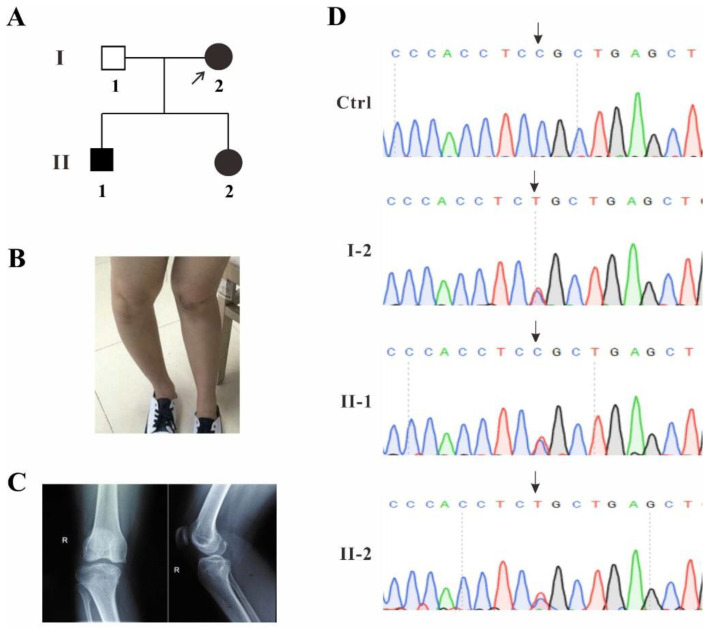
Analysis of a novel *TBX4* mutation. (**A**) The pedigree of the family with three patients and the proband is indicated by arrow. (**B**) Leg appearance photo of the proband I-2. (**C**) X-ray report of the proband I-2. (**D**) Sanger sequencing results from the three patients and a healthy control, the mutation sites are indicated by arrows.

A family with SPS clinical manifestations was recruited from Fujian Province, China, and the blood samples from three patients and one healthy volunteer were collected (Figure 1A). Proband I-2 displayed mild genu valgum and genu recurvatum, which was suspected to be caused by the dislocation of the small patella (Figure 1B). The patient’s right leg was examined by X-ray DR (Digital Radiography). The report showed that the knee joint was abnormal and the patella was displaced laterally (Figure 1C).

In order to identify whether or which gene mutation is disease-causative in this family, the genomic DNA of the proband I-2 was extracted from the blood and whole-exome sequencing was performed. The *TBX4* c.1241C>T (p.P414L) was identified to be the probably disease-causing mutation. Gene co-segregation was confirmed by Sanger sequencing for individuals I-2, II-1 and II-2 in this pedigree and heterozygous single base substitution in exon 8 of *TBX4* (c.1241C>T, p.P414L) was identified in all three patients (Figure 1D), which was not found in several human mutation databases such as HGMD, Clinvar, gnomAD, 1,000 Genomes, as well as the available literature, and this mutation was predicted to be disease-causing by Mutation Taster (disease causing, score: 0.9999; https://www.mutationtaster.org/).

### 2.2. TBX4 Plasmids Construction and Mesenchymal Stem Cells (MSCs) Cell Lines Screening

**Figure 2:**
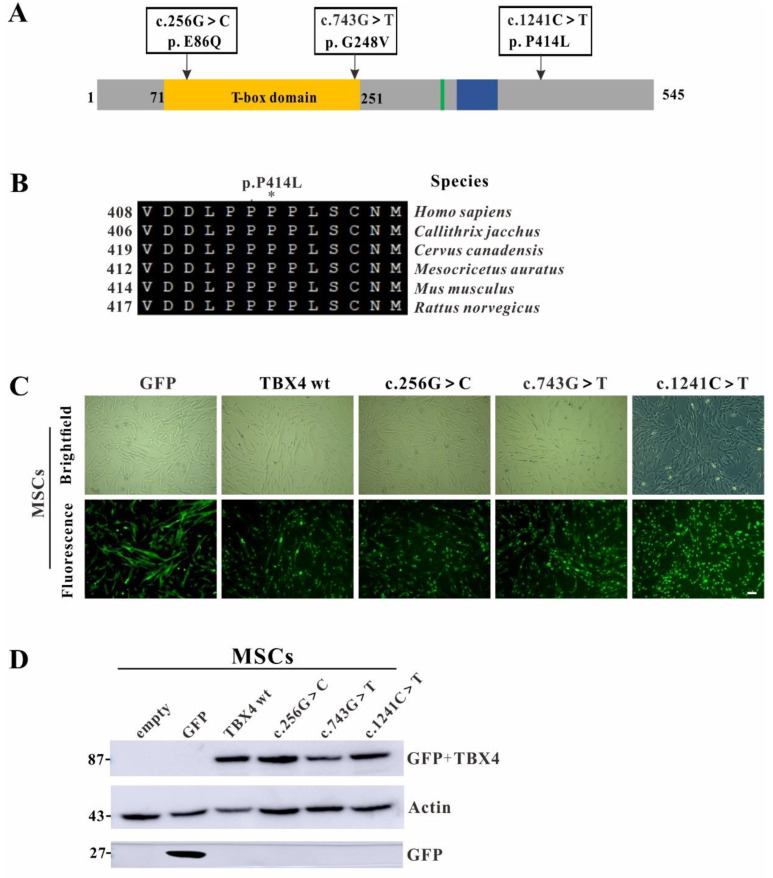
Generation of mesenchymal stem cells (MSCs) cell lines with stable overexpression of either *TBX4*^wt^ or *TBX4*^mt^. (**A**) Schematic of the secondary structure and functional domains of the TBX4 protein. The distribution of three mutations in TBX4 are indicated by arrow. The T-box domain, nuclear localization signal and regulatory interaction domain are shown in yellow, green and blue, respectively. (**B**) Conservation of the 414th amino acid in TBX4 protein among different species. NCBI accession numbers are: *Homo sapiens*: NP_001308049.1; *Callithrix jacchus*: XP_035156592.1; *Cervus canadensis*: XP_043337426.1; *Mesocricetus auratus*: XP_040605335.1; *Mus musculus*: NP_035666.2; *Rattus norvegicus*: NP_001100504.1. (**C**) Brightfield and immunofluorescence images of the overexpressed GFP fusion proteins in MSCs. Bar: 200 mm. (**D**) Western blot analysis for stable overexpression MSCs lysates. The molecular weights of expected band are 87 KDa with TBX4 (60 KDa) and GFP (27 KDa) in total.

In order to comprehensively understand the pathogenesis of this novel *TBX4* missense mutation, two previously reported mutations c.743G>T (p.G248V) and c.256G>C (p.E86Q) localized in T-box DNA binding domain were included for a joint study (Figure 2A). The *TBX4* c.743G>T (p.G248V) mutation has been identified as a *de novo* mutation from a SPS family and predicted to disrupt DNA binding activity partially or completely. The *TBX4* c.256G>C (p.E86Q) mutation has been identified from a 1-day-old deceased newborn, with severe diffuse developmental lung disorder exhibiting features of acinar dysplasia for the first time [7,20]. The functions of these two mutations have not been characterized. To identify the evolutionary conservation of the altered amino acid, multiple sequences alignment was performed. The result showed that the impaired amino acid residue P414 was highly evolutionarily conserved among TBX4 proteins from different species, indicating this mutation was likely the causative mutation resulting in SPS (Figure 2B).

The main features of SPS are dysplasia of the patella and pelvis, which is considered to affect the development of hindlimbs [7]. MSCs can overexpress foreign genes without affecting its basic biological activity [21]. TBX4 protein is highly expressed in the atrium of the heart, the mesenchyme of the lung and trachea, and the limbs. Therefore, MSCs are the most suitable cell type for the pathogenic investigation on the *TBX4* mutations. All three mutations were introduced into GFP-tagged full-length *TBX4* wild-type. The MSCs were transfected by the pseudo-lentiviral particles containing either *TBX4*^wt^ or *TBX4*^mt^ and the transfection efficiencies were examined post 7-days’ consecutive drug selection. Fluorescence microscopy results showed that most cells are positive with GFP fluorescence signal (Figure 2C). Western blot analysis revealed that TBX4^wt^ or TBX4^mt^ GFP fusion proteins were highly expressed by GFP monoclonal antibodies detection (Figure 2D), indicating that MSCs with stable overexpression of either *TBX4*^wt^ or *TBX4*^mt^ were successfully generated.

### 2.3. TBX4 Mutations Affect MSCs Osteogenic Differentiation and Promote Cell Senescence

**Figure 3:**
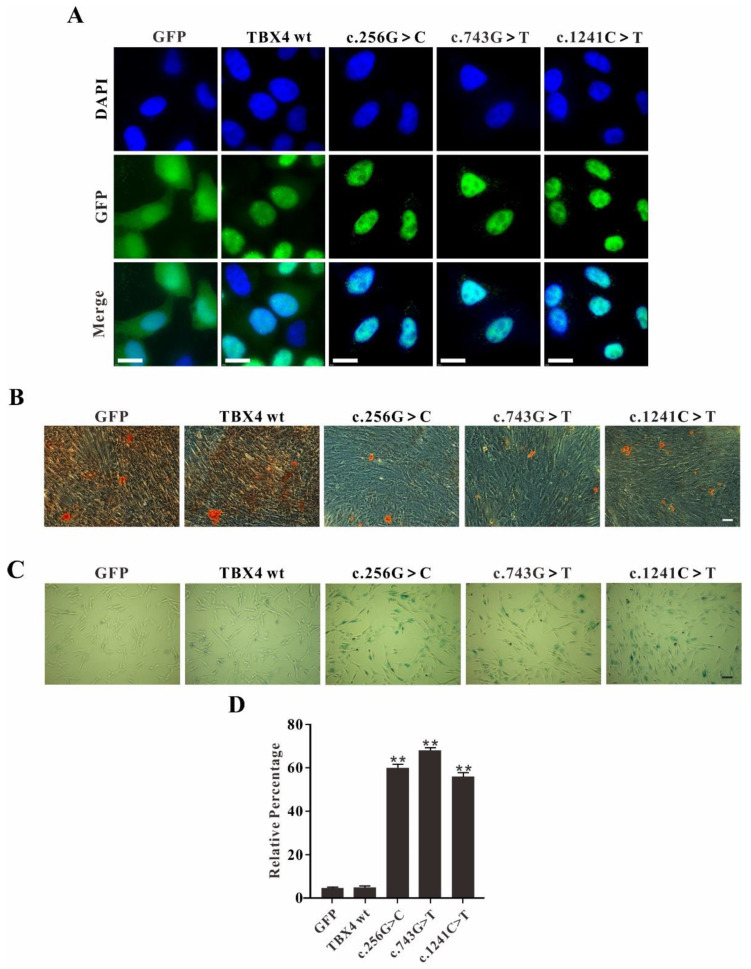
Analysis of *TBX4* mutations. (**A**) Localization of TBX4^wt^ or TBX4^mt^ GFP fusions in HeLa cells, and GFP was used as control. Bar: 25 μm. (**B**) Osteogenic differentiation of mesenchymal stem cells (MSCs) overexpression of either *TBX4*^wt^ or *TBX4*^mt^. The number of red calcium nodules correlates with the degree of cell differentiation. Bar: 200 mm. (**C**) Visualization of senescence associated β-galactosidase staining between wild-type and mutant TBX4 transfected cells. X-Gal was used as a substrate for β-galactosidase, and dark blue products inside cells were produced when catalyzed by aging-specific β-galactosidase. Examination for staining was performed after overnight incubation under bright field microscopy at 200× magnification. Bar: 200 mm. (**D**) Statistics of senescence associated β-galactosidase assay. The blue cells were counted, and the percentages of blue cells in total were calculated. SPSS was applied for statistics analysis (** *p* < 0.01).

As a transcription factor, TBX4 interacts with the promoter of the target gene in the nucleus. To identify whether the TBX4 mutants exhibiting altered localization inside the transfected cells, immunofluorescence study was performed. In GFP alone transfected cells, GFP signal was detected in both nucleus and cytoplasm. In both *TBX4*^wt^ and *TBX4*^mt^ transfected cells, the TBX4 fusions were uniformly expressed in the nucleus without significant difference suggesting that these mutations do not alter the translocation of TBX4 into the nucleus, no matter *TBX4*^wt^ or *TBX4*^mt^ (c.256G>C, c.743G>T and c.1241C>T) in this study (Figure 3A).

Since *TBX4* affects mesodermal differentiation and plays an important role in hindlimb branching morphogenesis, we asked whether *TBX4* mutations affect the osteogenic differentiation of MSCs. MSCs with stable overexpression of either *TBX4*^wt^ or *TBX4*^mt^ were used for the evaluation of osteogenic differentiation. In GFP and TBX4^wt^ overexpression cells, the numbers of red calcium nodules were significantly higher than that in all three TBX4^mt^ overexpression cells, indicating that the three *TBX4* missense mutations (c.256G>C, c.743G>T and c.1241C>T) affected the activities of MSCs in osteogenic differentiation (Figure 3B).

**Figure 4:**
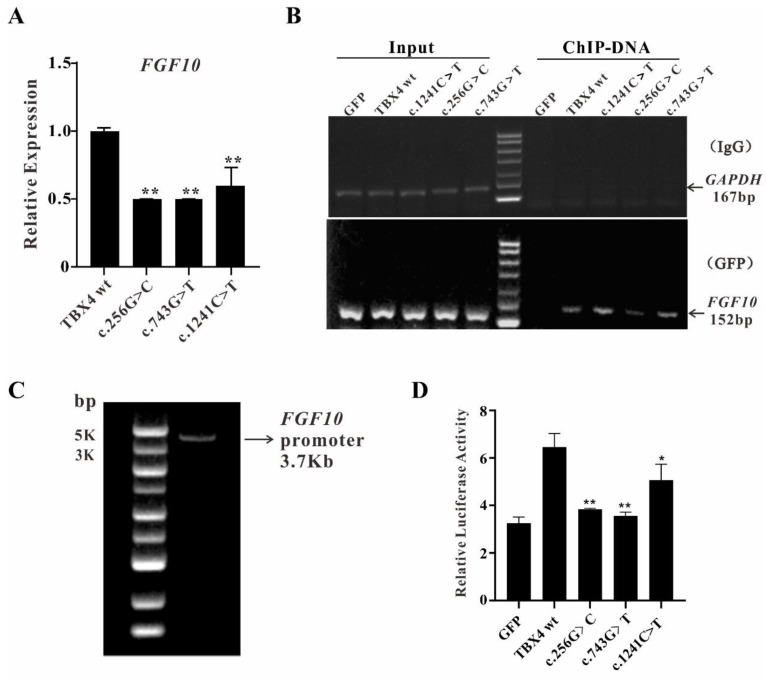
Investigations on TBX4-FGF10 pathway. (**A**) Quantification of the relative expression of *FGF10* mRNA in mesenchymal stem cells (MSCs) with stable overexpression of either *TBX4*^wt^ or *TBX4*^mt^ using qRT-PCR. Data represent the mean ± SD of three independent experiments performed in triplicate. (**B**) Chromatin immunoprecipitation (ChIP) assay for MSCs with overexpression of either *TBX4*^wt^ or *TBX4*^mt^. (**C**) The *FGF10* promoter with 3.7 kb was amplified by PCR. (**D**) Relative luciferase activities in cells co-transfected pGL3-*FGF10* with *TBX4* wild type or mutants, respectively (* *p* < 0.05; ** *p* < 0.01).

In order to further explore the cellular phenotypes caused from *TBX4* mutations, MSCs with stable overexpression of either *TBX4*^wt^ or *TBX4*^mt^ were subjected to β-galactosidase staining for cell senescence assay. The results showed that all the three missense mutations overexpressed MSCs exhibited more senescent cells compared to the *TBX4*^wt^ overexpression MSCs (Figure 3C,D). In GFP alone and *TBX4*^wt^ transfected cells, less than 10% of the cells were β-galactosidase positive; however, in *TBX4*^mt^ transfected cells, the positive cells were increased up to 60% (Figure 4E). Our results indicate that all three *TBX4* missense mutations result in the reduced activities of osteogenic differentiation and increased senescence of MSCs.

### 2.4. The Reduced Binding Efficiences of TBX4 Mutants to FGF10 Promoter

It was known that the TBX4-FGF10 pathway plays a key role in hindlimb development [22]. Since *TBX4* mutations affect cell differentiation and senescence, we are wondering whether the abnormal development of hindlimb is caused by an impaired TBX4-FGF10 pathway [16]. To test our hypothesis, qRT-PCR was performed to quantify the relative mRNA expression of *FGF10* in MSCs with overexpression of either *TBX4*^wt^ or *TBX4*^mt^. The results showed that the expression of *FGF10* is significantly decreased in all three missense mutations overexpressed MSCs, indicating that TBX4 wild-type is involved in the initiation or maintenance of *FGF10* expression, whereas mutations in *TBX4* lead to the reduced *FGF10* transcriptional activity (Figure 4A).

To further explore the binding abilities between TBX4 mutants and *FGF10* promoter, chromatin immunoprecipitation (ChIP) assays were performed using MSCs with overexpression of either *TBX4*^wt^ or *TBX4*^mt^. The ultrasonicated total DNA were indicated as input and the immunoprecipitated DNA by IgG or GFP antibodies were amplified using the control primers of *GAPDH* and the target gene primers of *FGF10*, respectively. The results showed that the *GAPDH* signals were only positive in the input samples, indicating that the treatment worked under our experimental condition. The *FGF10* signals are positive in all input samples and only the ChIP-DNA samples from MSCs with overexpression of either *TBX4*^wt^ or *TBX4*^mt^, suggesting that the TBX4 binding abilities with *FGF10* promoter were not disturbed by these three *TBX4* mutations (Figure 4B). The MSCs with overexpression of GFP only were used as negative control.

To find out whether the binding efficiencies of TBX4 mutants with *FGF10* promoter are impaired due to *TBX4* mutations, dual luciferase reporter system was applied. MSCs transiently expressing either *TBX4*^wt^ or *TBX4*^mt^ were generated for dual luciferase report assay. The *FGF10* promoter with the molecular weight about 3.7 kb was obtained by PCR (Figure 4C) and pGL3-*FGF10* plasmid-containing *FGF10* promoter was constructed successfully as well. The results showed that the dual luciferase activity was significantly higher in the cells co-transfected with pGL3-*FGF10* and TBX4 wildtype compared with GFP control group, indicating the system worked under our experimental condition. However, the dual luciferase activities were much lower in cells co-transfected with pGL3-*FGF10* and any TBX4 mutants (Figure 4D), suggesting that these *TBX4* mutations affect the binding efficiency of TBX4 with *FGF10* promoter resulting in the reduced expression of *FGF10*. 

## 3. Discussion

We reported a novel *TBX4* mutation c.1241C>T in a family with clinical SPS presentations, which is the second case from Asian population whereas the first case is a Japanese woman [23]. The identified *TBX4* c.1241C>T mutation in our study is mapped to the non-T-box region of TBX4 protein with proline replaced by leucine. Since both amino acids are non-polar amino acids, the polarity of the protein was considered to be unchanged due to this mutation. However, the hydroxylation occurs in the peptide chain to form 4-hydroxyproline, the substitution of proline to leucine may lead to the conformation change of the protein.

The vertebrate limb develops from a small bud of undifferentiated mesoderm cells encased in ectoderm. The key transcription factors Tbx4 (leg/hindlimb) and Tbx5 (wing/forelimb) have equivalent functions in bud formation by initiating a signaling cascade involving Wnts and fibroblast growth factors (FGFs) and by regulating recruitment of mesenchymal cells from the coelomic epithelium into the bud [4,24,25]. MSC belongs to pluripotent stem cells with continuous self-renewal and multidirectional differentiation potential (adipogenic, osteogenic, endothelial) [26]. Therefore, primary MSCs with the potential of osteogenic differentiation were firstly used to study the function of *TBX4* mutations in this study and the results showed that *TBX4* mutations c.1241C>T, c.256G>C and c.743G>T affect osteogenic differentiation and promote cell senescence.

It was reported that both *Tbx4*^−/−^ and *Fgf10*^−/−^ mice or chickens exhibited lung and limb dysplasia phenotypes [27,28] indicating that TBX4-FGF10 pathway may plays a role in the development of SPS. In human, both *TBX4* and *FGF10* pathogenic variants resulted in typical SPS clinical futures including alveolar dysplasia and abnormal hindlimb development [29,30]. Therefore, *FGF10* is an important downstream gene of TBX4, and the function of the TBX4-FGF10 pathway is crucial for the maintenance of physiological lung functions and the development of limbs [31,32]. Once mutation occurs, the pathological features appear due to the impaired transcription of *FGF10*.

**Figure 5:**
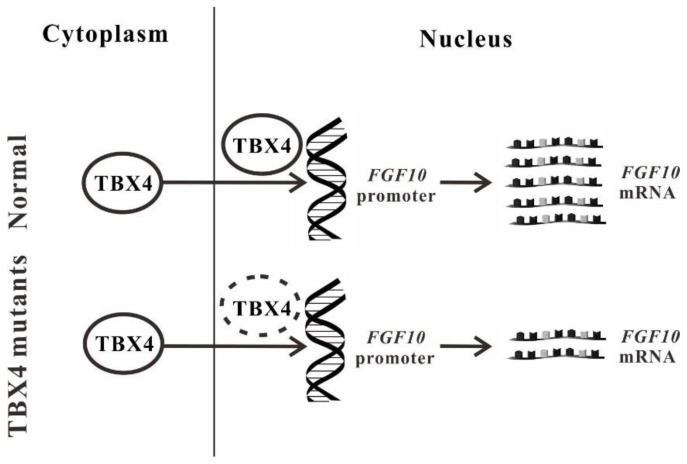
Schematic diagram of *TBX4* mutations resulting in small patella syndrome (SPS). Arrows indicate that *TBX4* mutations do not alter the translocation of TBX4 into the nucleus. The dashed oval indicates the reduced binding efficiency of TBX4 mutants with the *FGF10* promoter.

TBX4 is an important transcription factor. In this paper, qRT-PCR confirmed that all three *TBX4* mutations affected the expression of *FGF10*. Dual luciferase reporter assay is widely used for the investigations on gene transcription regulation and promoter transcription activity [33]. The cDNA of Renilla luciferase (Rluc) involved in the pGL3-TK vector is used as an internal reference to eliminate the differences in cell number or transfection efficiency [34]. The results demonstrated that *TBX4* mutations reduced the expression of *FGF10*. Considering that *FGF10* may be not the unique target gene of TBX4, ChIP-seq will be a powerful tool for exploring more target genes of TBX4 [35]. Taken together, our study indicates that *TBX4* mutations do not alter the translocation of TBX4 into the nucleus and the binding ability with *FGF10*, but exhibit less efficiencies to initiate the transcription of *FGF10* resulting in the abnormal biological processes (Figure 5).

More and more studies have revealed that spatial- and temporal-coordinated signaling pathways, mediated by transcription factors, control the stereotypic features and differentiation in limb development. Some transcription factors act as the major regulators for cell differentiation orientations and developmental patterns by regulating the expression of downstream gene [36]. The TBX4-FGF10 pathway plays a central role for both limb and lung development. Mutations either in transcription factor gene or the transcription factor binding site of the target gene are major causes of human diseases. The transcription factor is involved in the expression regulation of downstream gene, therefore, understanding either the enhancement or the suppression effect on the expression of target gene is the fundamental information for the manipulation of gene expression. Multiple transcription factors must work together to regulate the pathway, but the details of the interactions are mostly unknown [37]. The abnormalities of the TBX4-FGF10 pathway caused by *TBX4* missense mutations have been illustrated in this paper. However, the other pathways related to TBX4 have not been detected, no matter the upstream or the downstream/target gene. Recently, *Tbx4* has been identified as a novel transcriptional activator of short stature homeobox gene *Shox2* during murine fore- and hindlimb development. Tbx4 is also regulated by Shox2 specifically in the forelimb bud possibly via a feedback mechanism [38]. Further, a gene regulatory network including Tbx4, Pitx1 and Isl1 has been reported in the hindlimb bud establishment and the key differences of the pathway in initiating the formation of hindlimb or forelimb were characterized as well [39]. Although our results reveal the molecular mechanism of *TBX4* mutations resulting in SPS through TBX4-FGF10 pathway, the other pathways including the downstream gene or the interaction partners should be investigated further.

## 4. Materials and Methods

### 4.1. Patients

A 23-year-old female patient with SPS phenotype was recruited from Fuding City, Fujian Province, China. The patient was subjected to clinical and physical examinations and all her medical records were reviewed and evaluated. The study was approved by the Ethics Committee of Shanxi University (approval number: SXULL2019068), and the informed consent form was obtained from the patient.

### 4.2. Whole-Exome Sequencing and Sanger Sequencing

Whole-exome sequencing was performed by Veritas genetics (Hangzhou, China). Whole-exome enrichment was performed using SureSelect XT Target Enrichment System (51 Mb) according to the manufacturer’s protocols (Agilent, Santa Clara, CA, USA). Captured libraries were loaded onto the HiSeq 2500 platform (Illumina, San Diego, CA, USA). An average sequencing depth of 100-fold was achieved. Paired-end sequences were first aligned to the NCBI human reference genome (hg19), and the reads were mapped by Burrows-Wheeler Alignment (BWA) v0.7.12. To identify potential mutations, we performed local realignments using the Genome Analysis Toolkit (GATK). Variants were functionally annotated and filtered using our cloud-based rare disease NGS analysis platform with build in public databases (dbSNP, OMIM, ESP, Clinvar, 1000 Genomes, and ExAC) and HGMD professional database. Exonic sequence alterations and intronic variants at exon-intron boundaries, with unknown frequency or minor allele frequency (MAF) <1% and not present in the homozygous state in those databases were retained. Candidate variants through exome sequencing were confirmed using Sanger sequencing. For Sanger Sequencing, genomic DNA was extracted from 200 μL blood of the patients using TIANamp Genomic DNA Kit (TIANGEN Biotech, Beijing, China). The full-length *TBX4* DNA sequence (NM_018488.3, Genebank ID: 9496) was obtained from the NCBI website and Primer 5 software was used to design the primers. PCR was performed using the extracted DNA as the template and the PCR products were subjected to sequencing after agarose gel electrophoresis, extraction and purification of the amplified DNA fragments (Sangon Biotech, Shanghai, China). Primer sequence: *TBX4* forward: 5′-CGCCACCTGGACTTACCT-3′; *TBX4* reverse: 5′-CGGACCTGAGACTGGGAGA-3′.

### 4.3. Plasmids and Stable Cell Lines Construction

The full-length *TBX4* cDNA was purchased from Source BioScience (Nottingham, UK) and subcloned into the pEGFP-N1 and PLVX-IRES-Puro expression vector at multiclonal sites of XhoI and BamHI/NotI. The construction of PLVX-IRES-Puro vector containing *TBX4* cDNA (PLVX-TBX4^wt^-IRES-Puro) was verified by Sanger sequencing. The Site-directed mutagenesis kit was used to generate the *TBX4* mutants’ plasmids in the PLVX-TBX4^wt^-IRES-Puro vector according to the manufacturer’s instructions (ThermoFisher Scientific, Waltham, MA, USA, Cat. No. A13282). HEK293T cells were prepared at 50–70% confluence for transfection, the pMD2.G and psPAX2 packaging plasmids were co-transfected with the PLVX-IRES-Puro plasmids containing *TBX4* cDNA with the ratio of 1:5:5 using polyetherimide (PEI). The medium containing pseudo-lentiviral particles was collected at 24 h and 48 h post-transfection, respectively. The supernatants were purified by centrifugation at 1000× *g* and filtration through a 0.45 μm filter. The MSCs at 30-50% confluence in 12-well plate were infected with the supernatants containing pseudo-lentiviral particles and fresh medium at the ratio of 3:1. After 2 days, the drug selection with puromycin (1 mg/mL) was performed for 7-10 days to obtain stable transfected cells expression of either *TBX4^wt^* or *TBX4^mt^*. The primers used for mutagenesis are shown as follows: c.1241C>T forward: 5′-GACCTGCCCCCACCTCTGCTGAGCTGTAACATG-3′; c.1241C>T reverse: 5′-AGAGGTGGGGGCAGGTCGTCCACCCCAGACAC-3′; c.256G>C forward: 5′-TCCACGAGGCGGGCACCCAGATGATCATCACTAAG-3′; c.256G>C reverse: 5′-GGGTGCCCGCCTCGTGGAACTTCTTCCAGAGCT-3′; c.743G>T forward: 5′-CAACCCTTTTGCCAAGGTATTCCGGGGCAGTGATG-3′; c.743G>T reverse: 5′-ACCTTGGCAAAAGGGTTGTTCTCAATTTTCAGCTGG-3′.

### 4.4. Immunoflurorescence

The coverslips were placed in 6-well plate and transient transfection was performed with HeLa cells cultured in DMEM (Boster, Wuhan, China) with 10% fetal bovine serum (Gibco, ThermoFisher Scientific, Waltham, MA, USA) and 1% penicillin/streptomycin, and grown with 5% CO_2_ at 37 °C for 2 days until 60% confluence. The cells were fixed by the addition of 4% paraformaldehyde at 37 °C for 10 min, and then washed with PBS for three times. The cells on coverslips were stained with DAPI (Solarbio, Beijing, China) for 30 min and mounted on the glass slide for visualization. Slides were viewed and images were acquired using DeltaVision with a 100x objective (DeltaVision Elite, GE) after PBS washing.

### 4.5. Osteogenic Differentiation of MSCs

The MSCs overexpression of either *TBX4*^wt^ or *TBX4*^mt^ were seeded at the density of 5 × 10^4^ cells/cm^2^ in a 12-well plate pre-coated with 0.1% gelatin. When the cell density reached up to 70%, the medium was replaced by 2 mL fresh basal medium for osteogenic differentiation (Cyagen Biosciences, Suzhou, China, Cat. No. HUXUC-90021) containing 10% FBS, 100 IU/mL penicillin-streptomycin, 0.1 μM dexamethasone, 10 mmol/L β-Glycerophosphate, 0.1 mmol/L ascorbate and 10 mmol/L glutamine. The cells were differentiated for 18 days, and stained with alizarin red. Cells were visualized under bright field microscopy at 200× magnification.

### 4.6. Senescence Associated β-Galactosidase Assay

The MSCs overexpression of either *TBX4*^wt^ or *TBX4*^mt^ were seeded in 6-well plate and cultured in DMEM with 10% FBS (Gibco, ThermoFisher Scientific, Waltham, MA, USA) and 1% penicillin/streptomycin and grown until 70% confluence with 5% CO_2_ at 37 °C. The cells were washed with PBS and fixed with 3% PFA (5 min, RT). After twice washing with PBS, cells were incubated in a CO_2_-free incubator at 37 °C with the addition of freshly prepared senescence-associated-Gal (SA-Gal) staining solution (Solarbio, Beijing, China, Cat. No. BC2580) for *β*-galactosidase assay. After overnight incubation, cells were visualized under bright field microscopy at 200× magnification.

### 4.7. qRT-PCR

Total RNAs from MSCs were extracted for reverse transcription (Takara, Dalian, China, Cat. No. RR047A). Specific primers for qRT-PCR were designed using Primer 5. The relative expression levels of the target genes were quantified using SYBR Green qRT-PCR kits (Yeasen, Shanghai, China, Cat. No. 11201ES08). SPSS was applied for statistics analysis. The primer sequences are shown as follows: *FGF10* forward: 5′-CAGTAGAAATCGGAGTTGTTGCC-3′; *FGF10* reverse: 5′-TGAGCCATAGAGTTTCCCCTTC-3′.

### 4.8. Chromatin Immunoprecipitation (ChIP)

ChIP experiments were performed according to the instructions of the manufacturer (Beyotime Biotechnology, Shanghai, China). Briefly, MSCs with the stable overexpression of either *TBX4*^wt^ or *TBX4*^mt^ were cultured until 90% confluence in a 10 cm dish with 10 mL medium, and cells were incubated for 10 min at 37 °C after the addition of 270 μL 37% formaldehyde (final concentration is 1%). The cross-linking of genomic DNA with proteins was terminated by the addition of 1.1 mL glycine solution (10×) with the incubation for 5 min at room temperature. After twice washing with pre-chilled PBS containing 1 mM PMSF, the cell pellets were fully lysed using SDS Lysis Buffer provided from the kits for 10 min on ice. Genomic DNA was fragmented by 10 s sonication for 3–4 times at a power of 50 W on ice. The purification of fragmented DNA was performed 3 times with phenol chloroform extraction. Fragmented DNA was analyzed by gel electrophoresis to ensure that fragments with 200 to 1000 bp were obtained before incubating with GFP antibodies which recognizes GFP-TBX4 fusion proteins. DNA pellets were resuspended in DNase-free water, and analyzed by PCR using the following cycling program: 95 °C for 3 min pre-denaturation; a second step for 20 s at 94 °C; 58 °C for 25 s; and 72 °C for 25 s, with 35 cycles in total. PCR products were tested by 2% agarose gel electrophoresis. Primers were designed at 2 kb upstream of *FGF10* transcription start site and *GAPDH* primers were provided from the kit, and sequences are shown as follows: *FGF10* forward: 5′-TGAATGGCTTCTTCTACTGG-3′; *FGF10* reverse: 5′-CCAACACTATTGATGCCACT-3′; *GAPDH* forward: 5′-TACTAGCGGTTTTACGGGCG-3′; *GAPDH* reverse: 5′-TCGAACAGGAGGAGCAGAGAGCGA-3′. 

### 4.9. Dual Luciferase Reporter Assay

*FGF10* promoter was obtained from the human genomic DNA by PCR. The fragment containing 3.0 kb upstream of *FGF10* transcription start site and 0.7 kb of 5′ UTR (3.7 k in total) was inserted into pGL3-basic vector to generate pGL3-*FGF10* plasmid. Reaction conditions were: 95 °C for 3 min pre-denaturation; a second step for 20 s at 94 °C; 58 °C for 25 s; and 72 °C for 4 min, which was repeated for 35 times. Dual luciferase reporter system was carried out as described previously [40]. Briefly, 5 × 10^4^ MSCs were seeded in 48-well plate in triplicate and settled for 12 h. The plasmids pRL-TK, pGL3-*FGF10*, GFP-TBX4-WT (*TBX4*^wt^) or GFP-TBX4-MT (*TBX4*^mt^) were co-transfected at a ratio of 1:1:10. At 36 h post-transfection, firefly luciferase and renilla signals were detected according to the instructions of the manufacturer (Dual Luciferase Reporter Gene Assay Kit, Promega, Madison, WI, USA, Cat. No. E1980) using a multifunctional microplate reader (BioTek, USA). The primers are shown as follows: *FGF10* promoter forward (KpnI): 5′-CGGGGTACCAGTCTGGGAGCAGGAGTAAA; *FGF10* promoter reverse (XhoI): 5′-CCGCTCGAGCCTATGATGTGCGTTTGACC.

### 4.10. Statistical Analysis

All data are presented as the mean ± SD from at least three separate experiments. The *p*-values were determined using the GraphPad Prism software 8 (GraphPad Software, Inc., La Jolla, CA, USA). *p* < 0.05 was considered as being significant.

## Data Availability

All primary data are available upon reasonable request.
